# Is Diet Perceived as a Cancer Risk Factor? Lay Perceptions in a Representative French Sample

**DOI:** 10.1002/cam4.70944

**Published:** 2025-05-15

**Authors:** Kristopher Lamore, Valentyn Fournier, Iris Cervenka, Bernard Srour, Jérôme Foucaud

**Affiliations:** ^1^ Institut National du Cancer (INCa) Boulogne‐Billancourt France; ^2^ Univ. Lille, CNRS, UMR 9193 ‐ SCALab ‐ Sciences Cognitives et Sciences Affectives Lille France; ^3^ Université Paris Cité, Laboratoire de Psychopathologie et Processus de Santé Boulogne‐Billancourt France; ^4^ Université Sorbonne Paris Nord and Université Paris Cité, INSERM, INRAE, CNAM, Center of Research in Epidemiology and StatisticS (CRESS), nutritional Epidemiology Research Team (EREN) Bobigny France; ^5^ Nutrition and Cancer Research Network (NACRe Network) Jouy‐en‐Josas France; ^6^ Université Sorbonne Paris Nord, Laboratoire Éducations et Pratiques de Santé UR 3412 Bobigny France

**Keywords:** cancer barometer, nutrition, perceptions, population‐based study, risk factor

## Abstract

**Background:**

The French Cancer Barometer is a survey performed at five‐year intervals investigating awareness of cancer risk factors, including dietary factors. The aims of this study were (1) to report the results of the survey concerning participants' awareness of diet/foods as cancer risk factors, (2) to describe the determinants of this awareness, and (3) to compare the results of the 2010, 2015, and 2021 surveys.

**Methods:**

A randomly selected representative sample of participants aged from 15 to 85 years (*n* = 4938) was surveyed in 2021. Questions concerned awareness of diet, specific foods, and behaviors as cancer risk factors.

**Results:**

More than 40% of the participants spontaneously identified diet as a cancer risk factor, and almost 92% considered it to be a cancer risk factor in a closed‐ended question. Some foods were considered by most participants to increase cancer risk (ultra‐processed food, processed meats, and red meat), whereas others (fruit and vegetables) were perceived as decreasing cancer risk or were thought either to decrease risk or to have no effect (organic foods and pulses). Demographic factors were found to be associated with risk factor perceptions and specific perceptions of cancer (i.e., having a cancer someday, being well informed) and behaviors (i.e., having consulted a general practitioner recently). Interestingly, the perception of diet/food as risk factors was greater in 2021 than in previous surveys.

**Conclusion:**

In addition to continuing efforts to target interventions for individuals with lower educational levels, efforts should also be made to target individuals who consult their general practitioner infrequently.

## 1 Introduction

Cancer is a highly prevalent disease, affecting about one in two people in the Western world at some point during their lifetime [1]. In 2020, the number of new cancer cases was estimated at more than 19 million, with almost 10 million deaths from cancer [2]. Cancer remains the main cause of premature death in developed countries, including France [3]. It is therefore crucial to identify risk factors for cancer and to raise awareness of cancer risk factors so as to reduce their influence.

Two types of cancer risk factors can be distinguished: endogenous (e.g., chronological age and genetic predisposition) and exogenous (e.g., environmental or lifestyle‐related factors) factors. Exogenous factors are thought to account for up to 55% of cancer cases [4]. Interventions addressing endogenous factors may not be effective, but exogenous factors can be targeted [5]. More precisely, lifestyle factors, such as tobacco consumption, alcohol, diet, physical activity, overweight, and obesity and are among the most important preventable cancer risk factors [5].

Regarding diet, according to systematic reviews and meta‐analyses by the WCRF & AICR (World Cancer Research Fund & American Institute for Cancer Research) [6], there is strong evidence of an association between certain nutritional factors (e.g., fruit and vegetables, wholegrains, fiber‐rich foods, and vitamin D supplements) and a significantly lower risk of developing several cancers, and between others (e.g., alcohol consumption, red meat, processed meats, and high doses of beta‐carotene‐based dietary supplements) and a higher risk of developing cancers. For other foods or dietary behaviors, several recent prospective studies providing epidemiological evidence for such risk associations have yet not been summarized in the framework of the WCRF expert groups. There are frequent media reports on factors that increase or decrease the risk of cancer, but there is little scientific evidence to establish recommendations concerning factors such as ultra‐processed foods [7], organic foods [8], fasting and restrictive diets [9], or nutritional circadian rhythms [10]. Dietary behaviors have thus been widely considered in public health policies for decades, and specific prevention strategies do exist (e.g., television and poster campaigns). For example, since 2001, the National Nutrition and Health Program has defined nutrition‐related objectives to improve the health status of the general population [11].

Given the evidence, understanding how people perceive diet as a cancer risk factor is crucial to increasing public awareness and to implementing effective health interventions and reducing cancer‐related disparities. Over the years, surveys have therefore been conducted to assess the awareness of cancer risk factors. Such awareness has consistently been found to be low over the years for all factors other than tobacco use [12, 13, 14, 15]. For example, in 2023 in the United Kingdom, only 31% of participants spontaneously identified a poor or unhealthy diet as a cancer risk factor [15]. Furthermore, disparities in cancer risk awareness exist between different demographic groups, with a potential impact on disparities in cancer outcomes [16]. However, the awareness of the population concerning the role of specific foods (e.g., red meat and vegetables) has not been explored in such population‐based surveys.

The Cancer Barometer [17, 18, 19, 20], a population‐based survey, is performed every 5 years in France to explore the awareness of cancer risk factors in the general population. Detailed questions are asked about diet and foods. The aims of this study were (1) to describe the awareness of diet/food as a potential risk or protective factors for cancer in the French population in 2021, (2) to identify demographic variables associated with the awareness of these links, and (3) to assess changes in this awareness relative to previous surveys (2010, 2015, and 2021).

## 2 Materials and Methods

### 2.1 Study Design

The Cancer Barometer is a national survey performed by the French National Cancer Institute and Santé Publique France. It is based on computer‐assisted telephone interviews with questions that remain consistent over time (2010, 2015, and 2021), and it investigates awareness (perceptions and knowledge) of risk and protective factors for cancer in the French population. Here, we consider only the questions relating to diet.

The complete methodology of the surveys is presented in more detail elsewhere [17, 18, 19, 20].

### 2.2 Population and Recruitment Methods

In 2021, French‐speaking residents with a telephone aged between 15 and 85 were invited to participate in the survey, regardless of their cancer history.

A list of potentially eligible participants was generated by a random digit dialing process, using number prefixes aligned with the blocks of numbers designated by the French regulatory authority. In instances in which multiple individuals shared the same phone number, the Kish method [21] was used to determine the eligible household member. This technique was used to ensure the sample was representative of the French population.

### 2.3 Procedure

An information letter was sent to all potential participants. These individuals were then contacted by telephone, and verbal consent was obtained. If the person was unavailable at the time of initial contact, an appointment was scheduled. In cases in which the telephone calls were inconclusive, at least 40 attempts were made to conduct the survey, with efforts spread across various moments of the day and week. When an eligible participant was successfully contacted and available, eligibility was confirmed before proceeding with the questionnaire. Interviewers used Computer‐Assisted Telephone Interviewing (CATI) Software to manage telephone calls, appointments, and overall survey progression. The participants' responses were collected anonymously and based on self‐reports.

### 2.4 Survey

The survey began with sociodemographic and general information‐related questions. Participants were asked about their age, sex, educational level, marital status, professional occupation, monthly family income in Euros, place of residence, body mass index, perceived state of health, and alcohol and tobacco consumption. Their perceptions of the risk of being affected by cancer 1 day, whether they had consulted a general practitioner during the last 12 months (yes/no), and personal and family history of cancer (yes/no) were also recorded.

Before questioning the participants about specific cancer risk factors, the following (1) open‐ended question was asked: “In your opinion, what are the three main causes of cancer?”. The participants were then asked 12 closed‐ended questions concerning the links between diet/foods and cancer risk factors:

(2) “Do you think that diet has a ‘very important’, ‘somewhat important’, ‘somewhat unimportant’ or ‘not at all important’ role in cancer development?”

(3 to 12) “In your opinion: can the frequent consumption of (‘fruit and vegetables’, ‘red meat’, ‘dairy products’, ‘processed meats’ (in French = “*charcuteries*”), ‘organic foods’, ‘wholegrains’ (in French = “*féculents complets*”), ‘pulses’ (in French = “*légumes secs*”), ‘dietary supplements’, ‘ultra‐processed foods’): (‘lower’, ‘increase’, or ‘has no influence on’) cancer risk?”

(13) “In your opinion, skipping meals or not eating for a period can (‘lower’, ‘increase’, or ‘has no influence on’) cancer risk?”

Participants were also asked (14) about how well‐informed they felt about the cancer risks associated with diet (“very well”, “rather well”, “rather poorly”, or “really poorly”).

The questions were repeated if the participant initially answered “don't know”. If they were still unable to provide a response, the answer was ultimately recorded as “don't know”.

With the exception of ultra‐processed foods, the food types addressed in questions 3–12 were not defined for the participants. Ultra‐processed foods were defined as “products that have undergone various processing methods and contain ingredients not typically used in home cooking, such as additives. Examples include reconstituted plant‐based steaks, sweetened beverages, chicken or fish nuggets, and powdered soups”.

### 2.5 Statistical Analysis

Statistical analyses were performed with SPSS 28 software. The data were first weighted (according to the number of eligible participants, number of mobile telephones in the same household, age, place of residence, size of the municipality of residence, educational level and the whether the participant lived alone or with other people) to ensure representativeness with respect to the French population. Descriptive statistics were then computed, and chi‐squared tests were performed to identify the variables for inclusion in logistic regression models. Logistic regression analyses were conducted to evaluate associations between sociodemographic factors and participants' perceptions of the influence of diet and foods on cancer risk. A stepwise approach was used to identify the explanatory variables for each regression model, with age and sex as forced variables.

For Questions (2) and (14), the responses “very important/very well” and “somewhat important/rather well” were combined, as were the responses “somewhat unimportant/rather poorly” and “not important at all/really poorly”, to obtain a clear result concerning the number of participants perceiving cancer risks linked to diet/foods and their level of information (well‐informed versus poorly informed).

Finally, we performed Chi‐squared tests to compare the results of this survey with those of previous Cancer Barometer surveys (2010, 2015, and 2021).

The percentages presented in Table 2 and the Tables S1–S3 presenting the results of logistic regression analyses correspond to the proportions of respondents within each of the categories who perceived diet to be a risk factor for cancer (or protective factor or has having no influence on cancer risk). These percentages do not add up to 100% across all categories because they are presented independently for each subgroup. For example, the percentages for “sex” indicate the proportions of men and women who think that the dependent variable does or does not play a role in cancer development.

## 3 Results

Full information about participant recruitment is presented in Table S1. The total sample consisted of 4938 individuals aged between 15 and 85 years, including 477 (9.6%) people with a history of current or prior cancer. Detailed sociodemographic information for the participants is presented in Table 1.

**TABLE 1:** Characteristics of the participants.

Variables	2010 (*n* = 3345)	2015 (*n* = 3508)	2021 (*n* = 4938)	*p*‐Value for χ^2^ test
	%	%		
Sex				***
Male	43.8	49.0	45.4	
Female	56.2	51.0	54.6	
Age (years)				***
15–34	30.6	32.1	21.8	
35–44	20.8	17.5	14.9	
45–54	18.7	17.7	18.3	
55–64	19.2	15.9	19.3	
65–75	10.7	16.8	18.8	
76–85	—	—	6.9	
Monthly income (€/CU^1^)				***
€0–1100	27.4	31.6	25.1	
€1101–1800	34.4	34.7	31.6	
€1800	30.0	25.1	33.7	
Don't know/Declined to answer	8.2	8.6	9.7	
Education level				***
< High school	46.4	53.0	34.2	
High school	19.7	19.1	20.9	
Postsecondary	33.9	27.9	44.6	
Missing data	—	—	0.2	
Occupation				***
Employee	22.8	25.6	26.2	
Working class	17.4	23.8	14.4	
Tradesperson, storekeeper, self‐employed worker, or farmer	6.1	7.2	9.0	
Intermediate occupations	28.3	19.2	27.6	
Higher occupations	21.0	19.1	21.0	
Other, non‐workers	4.4	5.3	1.7	

*Note:* ***p < 0.001.

We investigated changes in awareness of diet/foods and cancer risk factors in the French population by comparing the data for this survey with those collected in 2010 and 2015. In total, 3345 and 3508 people aged between 15 and 75 years who had never had cancer were included in the studies in 2010 and 2015, respectively. In the 2021 sample, 4458 people aged between 15 and 75 years who had never had cancer were included.

### 3.1 Perceived Degree of Information and Perception of Diet as a Risk Factor in 2021

**FIGURE 1:**
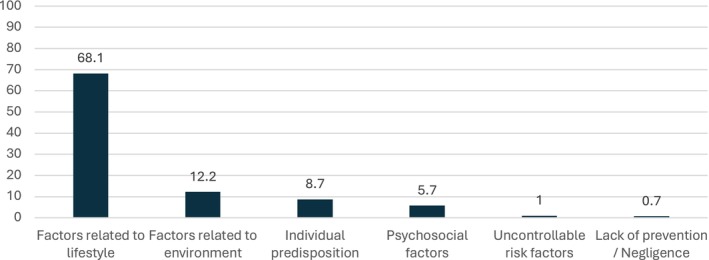
Main causes of cancer perceived by the participants in 2021. Factors related to lifestyle behaviours gathered: tobacco consumption (24.0%), diet/nutrition (15.3%), alcohol consumption (14.5%), hygiene and lifestyle (9.7%), lack of physical activity (2.5%), sun exposure (1.4%), overweight and obesity (0.8%), and drug consumption (0.6%). Factors related to environment gathered: pollution (5.8%), environment (4.3%), chemical and toxic products (1.7%), and professional exposure (0.8%). Individual predisposition corresponds to genetic mutations and heredity. Psychosocial factors correspond to words like “stress”, “emotional shock”, “anxiety”, “depression”, “mental illness”, or “distress”. Uncontrollable risk factors correspond to words like “bad luck” or “chance”.

Overall, 55.9% of participants considered themselves to be well informed about the role of diet as a risk factor in cancer. However, in an open‐ended question about the main causes of cancer (see Figure 1), only 39.9% of participants identified diet as one of the three main risk factors for cancer. Dietary factors were the second most frequently cited cause of cancer after tobacco (61.2%) and ahead of alcohol consumption (36.9%).

In a closed‐ended question, the vast majority of participants said that diet had an important role (91.8%). Univariate and multivariate analyses were performed on the answers given by the participants (see Table 2). No significant effects of sex, most recent occupation, region of residence, or educational level were observed in the multivariate analysis. The perception of diet as a risk factor for cancer was significantly greater among younger people, non‐smokers, people declaring themselves to be well informed, those who had consulted a general practitioner in the last 12 months, those who perceived themselves as at risk of cancer, and those with a personal or family history of cancer.

**TABLE 2:** Factors associated with the perception of diet as a risk factor (*n* = 4643).

	Univariate analysis		Multivariate analysis			
	%	*p*‐Value for χ^2^ test	OR	95% CI		*p*
				Lower	Upper	
Sex		ns				
Male (ref.)	92.20%		1			
Female	91.70%		0.9	0.7	1.1	
Age (years)		***				
15–24 (ref.)	89.90%		1			
25–34	94.30%		1	0.6	1.6	
35–44	92.10%		0.8	0.5	1.2	
45–54	91.60%		0.7	0.5	1.2	
55–64	93.30%		1	0.6	1.7	
65–74	93.90%		0.9	0.5	1.6	
75–85	86.10%		0.3	0.2	0.6	***
Occupation		***				
Employee (ref.)	91.60%		1			
Tradesperson, storekeeper, self‐employed worker, or farmer	94.00%		1.6	1	2.6	
Higher occupations	95.60%		1.4	0.9	2.3	
Intermediate occupations	94.00%		1.1	0.8	1.6	
Working class	87.70%		0.7	0.5	1	
Missing data	89.20%		0.7	0.5	1.2	
Education level		***				
< High school (ref.)	90.00%		1			
High school	91.80%		1	0.7	1.3	
Postsecondary	95.10%		1.3	0.9	1.8	
Smoking status		***				
No (ref.)	92.50%		1			
Yes	90.60%		0.7	0.5	0.9	***
Body mass index		***				
18.5–24.9 (ref.)	86.10%		1			
< 18.5	94.00%		0.4	0.3	0.7	***
25–29.9	91.50%		0.6	0.5	0.8	***
> = 30	88.70%		0.5	0.4	0.7	***
Region		**				
Pays‐de‐la‐Loire (ref.)	91.90%		1			
Paris region	91.60%		1.1	0.6	1.8	
Centre‐Val de Loire	92.10%		1.1	0.6	2.3	
Bourgogne‐Franche Comté	88.20%		1	0.5	1.9	
Normandie	92.10%		1.3	0.6	2.5	
Hauts‐de‐France	87.50%		0.7	0.4	1.3	
Grand‐Est	94.30%		1.5	0.8	2.7	
Bretagne	95.10%		1.5	0.7	3.1	
Nouvelle Aquitaine	93.40%		1.2	0.7	2.3	
Occitanie	94.20%		1.3	0.7	2.3	
Auvergne‐Rhône Alpes	92.30%		0.9	0.5	1.6	
Provence Alpes Côte‐d'Azur	90.30%		0.8	0.4	1.4	
Perceived level of information on the effects of diet on cancer risk		***				
Very well informed (ref.)	93.50%		1			
Somewhat well informed	93.30%		0.6	0.4	0.9	*
Somewhat poorly informed	90.90%		0.4	0.3	0.6	***
Very poorly informed	88.30%		0.4	0.2	0.7	***
Have you seen your general practitioner in the last 12 months?		***				
Yes (ref.)	92.60%		1			
No	89.10%		0.7	0.5	0.9	*
Do you have or have you had cancer?		ns				
No (ref.)	92.20%		1			
Yes	89.50%		1.5	1	2.2	*
Do you feel personally at risk of developing cancer during your life?		***				
Yes (ref.)	94.40%		1			
No	87.50%		0.5	0.4	0.7	***
Has at least one of your relatives had cancer?		***				
No (ref.)	82.70%		1			
Yes	93.00%		1.9	1.4	2.6	***

*Note:* For this analysis, data from 4643 of the 4938 participants were included, the rest being missing data. The percentages presented in the univariate analysis reflect the proportion of respondents within each category perceiving diet as a risk factor for cancer. For example, the percentages for “sex” indicate the proportion of men and women respondents who think that diet has a “very important”, or “somewhat important”, role in cancer development. These percentages do not add up to 100% across all categories because they are presented for each subgroup independently.

Abbreviations: CI, confidence interval; OR, odds ratio; ref., reference.

* *p* < 0.05.

** *p* < 0.01.

*** *p* < 0.001.

### 3.2 Perception of the Links Between Food Types and Cancer Risk in 2021

Some foods were considered by most of the participants to increase the risk of cancer (88.2% for ultra‐processed food, 74.1% for processed meats, and 62.4% for red meat); some were seen to decrease cancer risk (62.0% for fruit and vegetables), and the perception was mixed for others, which were considered either to decrease cancer risk or to have no influence (organic foods and pulses). Most of the participants perceived the remaining types of food as having no influence on cancer risk (67.8% for dairy products, 58.1% for wholegrains, and 52.4% for dietary supplements). The detailed answers given by the participants are displayed in Table 3.

**TABLE 3:** Participants' perceptions of cancer risk factors in 2021.

Questions and response	%	%	%	%
	(b) can lower cancer risk	(b) can increase cancer risk	(b) has no influence	(b) I don't know
In your opinion, frequent consumption of (a) … (b) … (*n* = 4938)				
(a) Fruit and vegetables	**62.2**	2.7	34.5	0.7
(a) Dairy products	**12.7**	18	67.8	1.4
(a) Wholegrains	**36.6**	4.0	58.1	1.2
(a) Red meat	3.2	**62.4**	32.9	1.5
(a) Processed meats	2.5	**74.1**	22.4	1.0
(a) Dietary supplements	10.0	**33.6**	52.4	4.0
(a) Organic foods	48.5	1.5	48.8	1.2
(a) Pulses	43.3	1.7	53.9	1.3
(a) Ultra‐processed food	1.5	88.2	9.4	0.9
In your opinion, skipping meals or not eating for a period can (b) … (*n* = 2466)				
	8.3	18.3	72.1	1.2

*Note:* in bold type = factors known to lower or increase cancer risk [13].

Multivariate analysis revealed how perceptions of food and cancer risk varied with sex, age, most recent occupation, educational level, tobacco consumption, body mass index, region of residence, perceived level of information about diet and cancer risk, perceived risk of cancer, consultation of a general practitioner in the last 12 months, and personal and familial history of cancer.

Four logistic regression models are presented in Table S2 for foods known to be associated with cancer risk (fruit and vegetables, wholegrains, red meat, and processed meat). In the logistic regression analysis, the consumption of fruit and vegetables and that of wholegrains were considered as protective factors, whereas the consumption of red meat and that of processed meat were considered as risk factors. As dairy products have been associated with both a lower risk of colorectal cancer (strong evidence) and a higher risk of prostate cancer (limited evidence) [6], we decided to consider them as “having no influence” in the logistic regression analysis.

Perception of the impact of red meat consumption on cancer risk was greater among men than among women, whereas perception of the effect of wholegrain consumption on cancer risk was greater among women. An impact of wholegrain consumption on cancer risk was perceived more frequently among younger participants (15–24 years old vs. 25–34 years old), whereas an effect of red meat and processed meat consumption on cancer risk was most frequently perceived among older people (25–64 years old). The links between cancer risk and the consumption of fruit and vegetables or processed meat were more frequently perceived by individuals in higher or intermediate occupations, whereas the links between cancer risk and the consumption of wholegrains or red meats were more frequently perceived by the working class. Participants with higher levels of education had a greater perception of the impact of food consumption on cancer risk and nonsmokers perceived an impact of wholegrains, red meats, and processed meats on cancer risk more frequently than smokers. Significant associations were observed for body mass index (for all food types except fruit and vegetables) and for region of residence (all food types). Participants were significantly more likely to perceive risks/benefits of foods for cancer risk if they felt they were very well informed. Links between cancer risk and the consumption of red meats and processed meats were more frequently perceived by individuals who had consulted a general practitioner recently. Finally, the links between cancer risk and the consumption of fruit and vegetables, red meat, and processed meat were more frequently perceived by participants who considered themselves to be at risk of cancer and those who had a relative who had been diagnosed with cancer.

Five logistic regression models are presented in Table S3 for foods for which scientific evidence is inconclusive or insufficient for the formulation of cancer‐specific recommendations (dairy products, pulses, ultra‐processed foods, organic foods, and dietary supplements). The consumption of pulses and that of organic foods consumption were considered as protective factors in the logistic regression analysis, whereas the consumption of dietary supplements or dairy products was considered to have no influence on cancer risk. In terms of sex, the links between cancer risk and pulses and ultra‐processed foods were more frequently perceived by men, whereas women significantly more frequently perceived dairy products and dietary supplements as having no influence on cancer risk. For age, the perception of an impact of organic foods and pulses on cancer risk was greater among younger people, whereas an effect of ultra‐processed food, dairy products, and dietary supplement consumption on cancer risk was more frequently perceived among older people. The links between cancer risk and pulses, ultra‐processed foods, and organic foods were more frequently perceived by individuals in intermediate occupations than by the other occupational groups, whereas working‐class individuals were less likely than general employees to consider dairy products to affect cancer risk. Participants with a higher level of education had a greater perception of the impact of food consumption on cancer risk for all types of foods other than dairy products. Tobacco consumption also had an effect on the perception of cancer risk, with nonsmokers more likely to perceive an impact of pulse and organic food consumption on cancer risk. Body mass index and region of residence affected the perception of the cancer risks associated with all food types except pulses. Participants were more likely to perceive risk/benefit/absence of influence of pulses and dietary supplements on cancer risk if they felt very well informed; the opposite pattern was observed for ultra‐processed foods and dairy products (i.e., risk significantly more likely to be perceived by participants who felt somewhat poorly informed than by those who felt very well informed). For organic foods, no significant associations were observed. Links between cancer risk and the consumption of pulses, ultra‐processed foods, and organic foods were significantly more perceived by individuals aware that they are at risk of cancer, whereas dairy products were significantly more frequently perceived as having no influence on cancer risk by persons who did not feel particularly at risk of cancer. Finally, links between cancer risk and the consumption of ultra‐processed food and organic food were significantly more frequently perceived by participants who had consulted a general practitioner in the last 12 months and those with a relative diagnosed with cancer. However, participants who had not consulted a general practitioner in the last 12 months significantly more frequently perceived dietary supplements as having no influence on cancer risk.

### 3.3 Perception of the Link Between Skipping Meals and Cancer Risk in 2021

Skipping meals was perceived by 72.1% of the participants as having no influence on cancer risk (see Table 3). Multivariate analysis revealed how the perception of the impact of skipping meals on cancer risk varied with sex, age, occupation, education level, tobacco consumption, body mass index, region of residence, perceived level of information about diet and cancer risk, perceived risk of cancer, consultation of a general practitioner in the last 12 months, and personal and familial history of cancer (see Table S3). There was significantly greater perception of skipping meals as having no influence on cancer risk among young people (15–24 years old) (relative to older age groups: 55–64 and 65–74 years old), people in the “tradespersons, storekeepers, self‐employed workers, or farmers” socioprofessional category, those living in the *Pays de la Loire* region relative to the other five regions, and those without relatives diagnosed with cancer.

### 3.4 Changes in Perceptions of Diet as a Risk Factor for Cancer Between 2010, 2015, and 2021

**FIGURE 2:**
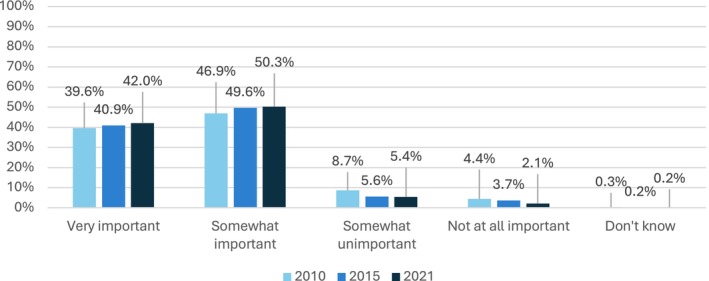
Changes between 2010 and 2021 of participants' perceptions of diet as having an important role in cancer risk (*p* < 0.001).

The results show a growing perception of diet as an important factor in the onset of cancer (see Figure 2) (*χ* ^2^(8) = 80.9; *p* < 0.001). When specific foods are considered separately, the participants had a stronger opinion in 2021 than previously. The proportion of “don't know” responses tended to decrease over time. Conversely, participants had a greater tendency to state that specific foods had no influence on cancer development (except for red meat). Finally, participants seemed to be more aware of the protective or deleterious role of some foods, notably for fruit and vegetables, red meat, and processed meat (see Table 4).

**TABLE 4:** Changes in participants' perceptions of cancer risk factors between 2010 and 2021.

Response	Fruit and vegetables			Diff. 2010–2021	Diff. 2015–2021	*p*‐Value for χ^2^ test
	2010	2015	2021			
Lowers risk	**55.90%**	**58.40%**	**62.80%**	**+6.90%**	**+4.40%**	***
Increases risk	2.80%	3.90%	2.50%	−0.30%	−1.40%	
No influence	18.40%	29.70%	33.90%	+15.50%	+4.20%	
Don't know	23%	8%	0.80%	−22.20%	−7.20%	

Response	Red meat			Diff. 2010–2021	Diff. 2015–2021	*p*‐Value for χ^2^ test
	2010	2015	2021			
Lowers risk	4.10%	6.20%	3.20%	−0.90%	−3.00%	***
Increases risk	**30.20%**	**42.70%**	**62.90%**	**+32.70%**	**+20.20%**	
No influence	16.40%	33.80%	32.50%	+16.10%	−1.30%	
Don't know	49.40%	17.30%	1.30%	−48.10%	−16.00%	

Response	Dairy products			Diff. 2010–2021	Diff. 2015–2021	*p*‐Value for χ^2^ test
	2010	2015	2021			
Lowers risk	**7.60%**	**11.90%**	**12.30%**	**+4.70%**	**+0.40%**	***
Increases risk	5.10%	13.90%	18.90%	+13.80%	+5.00%	
No influence	30.00%	52.90%	67.40%	+37.40%	+14.50%	
Don't know	57.30%	21.30%	1.30%	−56.00%	−20.00%	

Response	Processed meat			Diff. 2010–2021	Diff. 2015–2021	*p*‐Value for χ^2^ test
	2010	2015	2021			
Lowers risk	2.30%	2.30%	2.50%	+0.20%	+0.20%	***
Increases risk	**47.40%**	**62.20%**	**73.90%**	**+26.50%**	**+11.70%**	
No influence	8.40%	21.50%	22.60%	+14.20%	+1.10%	
Don't know	42.00%	13.90%	1.00%	−41.00%	−12.90%	

*Note:* In bold type = factors known to lower or increase cancer risk [13].

Abbreviation: Diff., difference.

*** *p* < 0.001.

## 4 Discussion

The French Cancer Barometer is original in that it surveys the awareness of cancer risk factors in a representative sample of the French population every 5 years. The results of the 2021 survey show an increase in awareness of the cancer risk factors related to diet. This survey allows a fine‐tuned description of the perception of diet/foods as cancer risk factors and the links between such perception and sociodemographic variables. The main finding is that most of the participants perceived diet as an important factor in cancer onset (with a closed‐ended question), whereas specific foods were not always perceived as cancer risk factors. However, when asked an open‐ended question about the main causes of cancer, fewer than one in two of the individuals questioned mention diet as one of the three main risk factors for cancer. Nevertheless, awareness of diet as a cancer risk factor has increased over the years in the French population.

### 4.1 Perception of Diet as a Determinant Factor in Cancer Risk

Since 2010, there has been a marked increase in the perception of diet as an important factor in cancer development. Indeed, more than 40% of the French population spontaneously considers diet to be a crucial risk factor for cancer. In the United Kingdom [15], 31% of the population spontaneously identified diet as a risk factor for cancer. By contrast, when closed‐ended questions were used, almost 92% of the participants in our study identified diet as a risk factor for cancer, as did almost 81% of a Spanish population sample in a similar survey [13]. This suggests that the French population may be more aware of this risk factor than other Western European countries. The public health policies relating to diet in individual countries may influence these results and studies are required to assess their impact.

The factors influencing perception changed between 2010 and 2021. In 2021, various factors were found to be associated with differing perceptions of the role of diet in cancer development. These factors included age, tobacco consumption, body mass index, considering oneself well‐informed about cancer risks related to diet, having consulted a general practitioner in the last 12 months, having a personal or family history of cancer, and perceiving a personal risk of developing cancer 1 day. Interestingly, by contrast to the findings of the surveys in 2010 and 2015 [22, 23], occupation and monthly income were not predictive of the perception of these risks in 2021. Sex also appeared to have no significant influence on the perception of diet as a cancer risk factor in 2021. In previous years, such as 2010 and 2015, women were more likely than men to perceive diet as having a significant role in cancer development. The absence of a significant difference between the sexes in 2021 may be attributed to better awareness and adherence to recommendations among men, or a growing interest in these factors in both sexes. No study has yet explored this aspect in detail. Further studies should explore the differences between the sexes in the appropriation of health recommendations and behavior change. The hypotheses that men have improved their understanding of recommendations, or that interest in these factors in both sexes has increased, could be tested.

### 4.2 A Heterogeneous Perception of Protective Factors

The protective role of specific foods was not always perceived by the participants in our study. Fruit and vegetables were seen as protective by 62% of participants, whereas wholegrains were perceived as protective by only 36.6% of the participants. We found a trend towards an increasing perception (+6%) of the protective role of fruit and vegetables against cancer since 2010 [22, 23], especially among individuals with a higher education level and those in higher level occupations. In the United States, fruit and vegetables are less frequently identified as protective, with this association perceived by only 42% of the individuals surveyed [24]. However, these results may be explained by methodological factors in the survey (i.e., the use of open‐ or closed‐ended questions).

The perception of the protective effects of consuming wholegrains was lower, potentially due to the recent nature of the recommendations and public health campaigns addressing this point, dating from 2019 in France [11]. Similar results were obtained in the United States [24], with only 38% of Americans perceiving the benefits of a high‐fiber diet. In France, wholegrains were the topic of a campaign broadcast three times since 2019. However, it should be noted that these campaigns talk about general health benefits and not specific benefits for cancer prevention, whereas scientific studies have shown that wholegrain consumption protects against colorectal cancer [6].

### 4.3 A Strong Perception of Cancer Risk Factors

The perception of processed meats and red meat as risk factors for cancer increased significantly from 31.2% and 62.9%, respectively, in 2010 to 47.4% and 73.9%, respectively, in 2021. Several public reports and scientific publications reporting links between the addition of nitrate salts to processed meats and cancer risk [25] have increased the awareness of this issue in the French media and the French population over this period. In other countries, a red meat is also perceived by a majority of individuals as representing a cancer risk.

### 4.4 Factors for Which Scientific Evidence Is Inconclusive or Insufficient for the Formulation of Cancer‐Specific Recommendations

A literature review identified about 50 international studies showing associations between the consumption of ultra‐processed foods and a higher risk of developing chronic diseases [26]. In our study, 88.2% of the participants perceived ultra‐processed food as a risk factor for cancer, possibly due to the considerable mediatization of these specific foods, in light, particularly, of the results of the French national “NutriNet‐Santé” cohort on the topic [27], the existence of recommendations to reduce the consumption of ultra‐processed foods [6], and the development of mobile apps (e.g., Open Food Facts, ScanUp) enabling consumers to identify ultra‐processed foods while shopping.

Almost half our participants (48.5%) considered organic foods to be protective against cancer, or to have no influence on cancer development (48.8%). This result may be due to recent epidemiologic studies suggesting associations between the consumption of organic foods and cancer risk [8]. These associations require further replication to obtain a high level of scientific evidence.

About one in three participants perceived dietary supplements as a risk factor, whereas 52.4% believed they had no influence on cancer risk. It should be noted that the question in the survey referred only to “dietary supplements” as a whole, without distinguishing between different types of supplements. To our knowledge, no other study has explored perceptions of this potential risk factor. Dietary supplements are products designed to complement the diet and provide nutrients that may not be consumed in sufficient quantities through food alone. These nutrients can include vitamins, minerals, amino acids, fatty acids, fiber, enzymes, probiotics, and other bioactive substances, and they are typically available in various forms, such as tablets, capsules, powders, liquids, and gummies. There is strong evidence that certain supplements, such as high‐dose beta‐carotene, increase the risk of lung cancer, particularly among smokers [6], but other supplements, such as calcium supplements (consumption at doses > 200 mg per day) have been linked to a probable decrease in the risk of colorectum cancers or to a limited suggestive decrease in the risk of colorectal cancers for multivitamins and vitamin D supplements [6]. For other supplements, such as vitamins A, B6, B9, B12 and E, selenium, zinc and omega‐3, the WCRF and AICR report [6] did not provide any results. Nonetheless, mixed results are often reported regarding the protective role of these dietary supplements. These risks are not widely discussed in the popular health discourse, which may be reflected by the responses obtained in our survey. However, the question asked was general. Future surveys should investigate in more detail the perceptions of the population regarding the potential impact of specific dietary supplements on cancer risk. In addition, official nutritional recommendations stress that the consumption of these supplements is unnecessary in the absence of a medical prescription, provided that the diet is well balanced [11]. It is recommended to meet nutritional needs through diet alone and not to use dietary supplements for cancer prevention purposes [6].

The evidence for dairy products is mixed. Milk and dairy products are known to protect against breast and colorectal cancers (limited‐suggestive decrease and probable decrease in risk, respectively), but they may increase the risk of prostate cancer (limited‐suggestive increase in risk) [6]. This may explain why only 12.7% of our participants perceived the consumption of dairy products to be protective against cancer, whereas 67.8% believed they had no influence on cancer risk. In addition, concerns about animal welfare, environmental issues, lactose intolerance, and changing dietary recommendations (see websites such as BE Vegan or Peta France) may have muddled the public perception of the benefits of dairy products and their risks to health.

Pulses were perceived as having no influence on cancer risk by most of the participants, but 43.3% perceive them as protective against cancer. This perception may be linked to recommendations formulated. Indeed, in France, public health agencies recommend eating pulses at least twice a week due to their plant protein content and their richness in fiber [28]. However, the available scientific data are insufficient for definitive conclusions to be drawn as to whether pulse consumption does or does not protect against cancer. It is, therefore, important to continue studying changes in the perception of pulses as a cancer risk factor.

Finally, regarding the practice of fasting (skipping meals or not eating for a certain period), most of those questioned (72.1%) felt that this practice had no influence on the risk of cancer. This result is consistent with published findings and a state‐of‐knowledge report published in 2017, which highlighted the lack of scientific evidence for cancer‐related benefits associated with fasting or restrictive diets [9].

### 4.5 Limitations and Future Research

The first limitation of this study is that the questions posed do not provide a precise understanding of the factors influencing the increase in the awareness of cancer risk in the French population. Certain variables that can influence participants' perceptions (e.g., psychological factors and personality) should be recorded. Secondly, as a means of mitigating potential bias in future studies, researchers should also ask participants how long it is since they last received information about diet and cancer risk or health. Thirdly, a limitation relating to participant recruitment is the absence of recorded information about ethnic or religious background, due to ethical restrictions on the collection of such data in France. Finally, the use of closed‐ended questions is also a limitation. Some of the food types considered were too general with different benefits or risks according to the type of cancer considered, as for dairy products and the consumption of dietary supplements. More specific or open‐ended questions could facilitate a better capture of the individuals' perceptions.

Future studies could include an ancillary study with a qualitative design to gain insight into participants' perceptions of cancer risk factors in relation to their behavior. This type of study could employ methods such as in‐depth interviews, focus groups, or open‐ended surveys to explore the nuanced ways in which individuals understand and respond to information about cancer risks. By examining the interplay between knowledge, attitudes, and practices, researchers would be able to identify the key barriers or motivators shaping health‐related behaviors. In addition, the attitudes and practices of individuals in relation to their perception, knowledge, and level of information would be investigated in mixed studies. For example, specific diets, such as vegetarian, vegan, raw vegan, and ketogenic diets, could be investigated, as some may include foods with a favorable (e.g., fruit, vegetables, and nuts) or unfavorable (e.g., ultra‐processed foods, saturated fats, and high consumption of red meat) nutrient profile. Other dimensions of food associated with cancer risk could also be studied, including the consumption of genetically modified organisms and the use of pesticides and packaging, for example. Such findings would be valuable for the design of targeted interventions and public health campaigns to increase awareness and promote preventive measures.

## 5 Conclusions

This study highlights the need to continue investigating the awareness of links between diet and cancer to identify, develop, and implement new approaches for public information campaigns. Indeed, sex was an important factor associated with individual perceptions in 2010 and 2015, but was less significant in 2021. In addition to the factors usually identified as linked to levels of awareness in the population (e.g., diplomas, education, and profession), new factors were identified in this study (i.e., having consulted a physician recently, individual perceived risk of cancer, having a relative with cancer), highlighting new groups for targeting. Future studies could focus on analyses of the levels of trust in official sources of information or the links between awareness of diet as a risk factor for cancer and dietary behaviors.

## Author Contributions

Kristopher Lamore: Conceptualization; methodology; writing – original draft; writing – review and editing. Valentyn Fournier: Software; formal analysis; writing – review and editing; writing – original draft. Iris Cervenka: Methodology; software; formal analysis; writing – review and editing; supervision. Bernard Srour: Methodology; writing – review and editing. Jérôme Foucaud: Conceptualization; methodology; writing – review and editing; resources; project administration.

## Ethics Statement

The authors state that they have followed the principles outlined in the Declaration of Helsinki for all human or animal experimental investigations. Informed consent has been obtained from the participants involved, and from the parents (if needed). The study was conducted in accordance with the French Data Protection Commission (CNIL), and all procedures were performed in accordance with the relevant guidelines and regulations. In compliance with French regulations, this survey does not require formal approval from an Institutional Review Board (IRB) or an Ethics Committee. Pursuant to Article L.1121‐1 of the French Public Health Code, observational studies that do not alter standard patient care or introduce additional procedures are exempt from mandatory ethics committee review. This study qualifies for such an exemption, notably as it involved the administration of anonymous questionnaires to the general population, with collection of anonymized demographic and survey response data. In conducting this study, we have adhered to the highest standards of ethical research. The design, implementation, and reporting of the study strictly follow guidelines to ensure transparency, accuracy, and reliability in the data collection and analysis process.

## Consent

All participants were fully informed about the purpose, scope, and potential outcomes of the study prior to participation. Informed consent was obtained from each participant, and participation was entirely voluntary. Measures have been taken to protect the privacy and confidentiality of all respondents, ensuring that no personally identifiable information is disclosed in the publication of this study.

## Conflicts of Interest

J.F. is the Head of the Humanities and Social Sciences, Epidemiology and Public Health Research Department at the INCa. I.C. is a research associate employed by the INCa. K.L. was a consultant hired by the INCa to perform this work. B.S. and V.F. declare no conflicts of interest.

## Supporting information


Tables S1–S3.

## Data Availability

Data available upon reasonable request.
