# Supplementary material for: Is Diet Perceived as a Cancer Risk Factor? Lay Perceptions in a Representative French Sample
Source: Cancer Med. 2025 May 15;14(10):e70944. doi: 10.1002/cam4.70944 (PMC12079020; doi:10.1002/cam4.70944)
Supplement: Supplementary file 1 — Tables S1–S3. [file CAM4-14-e70944-s001.docx]

**Supplementary Table 1.** Recruitment of participants.

|  | **2010** | **2015** | **2021** |
| --- | --- | --- | --- |
| **No. of phone numbers generated**  *Phone number not available or ineligible ^1^*  *Contactable numbers* | **25,706**  0  25,706 (100%) | **69,692**  38,204 (54.8%)  31,488 (45.2%) | **166,462**  142,159 (85.4%)  24 303 (14.6%) |
| **No. of eligible phone numbers** | **25,706** | **31,488** | **24,303** |
| *Not contacted ^2^*  *Contacted*  **No. of phone numbers contacted**  *Ineligible according to inclusion criteria*  *Declined to participate*  *No interview carried out ^3^* | 0  25,706 (100%)  **25,706**  15,854 (61.7%)  5,270 (20.5%)  576 (2.2%) | 12,989 (41.3%)  18,499 (58.7)  **18,499**  1,898 (10.3%)  9,839 (53.2%)  2,144 (11.6%) | 8,381 (34.5)  15,922 (65.5)  **15,922**  1,997 (12.5%)  6,528 (41.0%)  2,288 (14.4%) |
| *Dropped out*  *Missing information* | 278 (1.1%)  383 (1.5%) | 479 (2.6%)  375 (2.0%) | 171 (1.1%)  0 |
| *Final sample* | 3,345 (13.0%) | 3,764 (20.3%) | 4,938 (31.0%) |

Legend: 1. The telephone number generated was not attributed or ineligible (e.g., phone number of an association or a company); 2. No one was contacted after 40 attempts or the phone number was not used; 3. In some cases, an appointment was scheduled but it was impossible to reach the subject.

**Supplementary Table 2.** Factors associated with perception of the role of specific foods in cancer risk.

|  | **Fruit & vegetables (*n*=4638)**  **as a protective factor** | | | | |  | **Wholegrains (*n*=4611)**  **as a protective factor** | | | | |  |
| --- | --- | --- | --- | --- | --- | --- | --- | --- | --- | --- | --- | --- |
|  | **Model 1** | | | | |  | **Model 2** | | | | |  |
|  | **%** | **OR** | **Lower limit 95% CI** | **Upper limit 95% CI** | ***p*** |  | **%** | **OR** | **Lower limit 95% CI** | **Upper limit 95% CI** | ***p*** |  |
| **Sex** |  |  |  |  |  |  |  |  |  |  |  |  |
| Male (ref.) | 62.9% | 1 |  |  |  |  | 34.5% | 1 |  |  |  |  |
| Female | 62.1% | 0.9 | 0.8 | 1.1 |  |  |  | 1.2 | 1.1 | 1.4 | ** |  |
| **Age (years)** |  |  |  |  |  |  |  |  |  |  |  |  |
| 15-24 (ref.) | 62.4% | 1 |  |  |  |  | 34.1% | 1 |  |  |  |  |
| 25-34 | 68.7% | 1.2 | 0.9 | 1.6 |  |  | 33.7% | 0.7 | 0.6 | 1.0 | * |  |
| 35-44 | 61.1% | 0.9 | 0.7 | 1.2 |  |  | 36.5% | 0.9 | 0.7 | 1.1 |  |  |
| 45-54 | 60.5% | 1.0 | 0.7 | 1.3 |  |  | 34.8% | 0.9 | 0.7 | 1.2 |  |  |
| 55-64 | 64.1% | 1.2 | 0.9 | 1.6 |  |  | 42.2% | 1.2 | 0.9 | 1.6 |  |  |
| 65-74 | 63.4% | 1.1 | 0.8 | 1.5 |  |  | 44.6% | 1.3 | 0.9 | 1.7 |  |  |
| 75-85 | 53.9% | 0.8 | 0.6 | 1.1 |  |  | 31.6% | 0.7 | 0.5 | 1.0 |  |  |
| **Occupation** | | | | | |  |  |  |  |  |  |  |
| Employee (ref.) | 57.4% | 1 |  |  |  |  | 37.2% | 1 |  |  |  |  |
| Tradesperson. storekeeper. self-employed worker. or farmer | 57.1% | 0.9 | 0.7 | 1.2 |  |  | 34.6% | 0.9 | 0.7 | 1.2 |  |  |
| Higher occupations | 78.9% | 1.7 | 1.3 | 2.2 | *** |  | 45.3% | 1.2 | 0.9 | 1.5 |  |  |
| Intermediate occupations | 70.5% | 1.3 | 1.1 | 1.6 | * |  | 44.3% | 1.2 | 1.0 | 1.5 |  |  |
| Working class | 49.3% | 0.8 | 0.7 | 1.0 | * |  | 25.7% | 0.7 | 0.6 | 0.9 | ** |  |
| Missing data | 65.7% | 1.5 | 1.1 | 2.0 | ** |  | 32.4% | 0.9 | 0.6 | 1.1 |  |  |
| **Education level** |  |  |  |  |  |  |  |  |  |  |  |  |
| < High school (ref.) | 52.0% | 1 |  |  |  |  | 31.7% | 1 |  |  |  |  |
| High school | 67.3% | 1.6 | 1.4 | 1.9 | *** |  | 36.6% | 1.2 | 1.0 | 1.4 | * |  |
| Postsecondary | 75.3% | 2.0 | 1.7 | 2.4 | *** |  | 45.6% | 1.6 | 1.3 | 1.8 | *** |  |
| **Tobacco consumption** | | | | | |  |  |  |  |  |  |  |
| No (ref.) | 63.8% | 1 |  |  |  |  | 39.2% | 1 |  |  |  |  |
| Yes | 59.4% | 0.9 | 0.7 | 1.0 |  |  | 32.0% | 0.8 | 0.7 | 0.9 | ** |  |
| **Body mass index** | | | | | |  |  |  |  |  |  |  |
| 18.5-24.9 (ref.) | 61.8% | 1 |  |  |  |  | 30.7% | 1 |  |  |  |  |
| <18.5 | 65.2% | 0.9 | 0.6 | 1.2 |  |  | 39.9% | 0.7 | 0.5 | 0.9 | * |  |
| 25-29.9 | 61.2% | 0.9 | 0.8 | 1.0 |  |  | 34.5% | 0.8 | 0.7 | 0.9 | ** |  |
| >=30 | 58.4% | 0.9 | 0.8 | 1.1 |  |  | 36.8% | 0.9 | 0.8 | 1.1 |  |  |
| **Region** |  |  |  |  |  |  |  |  |  |  |  |  |
| Pays-de-la-Loire (ref.) | 63.9% | 1 |  |  |  |  | 36.4% | 1 |  |  |  |  |
| Ile-de-France (Paris region) | 67.0% | 1.0 | 0.8 | 1.4 |  |  | 41.0% | 1.1 | 0.8 | 1.5 |  |  |
| Centre-Val-de-Loire | 58.5% | 0.9 | 0.6 | 1.3 |  |  | 30.9% | 0.8 | 0.5 | 1.2 |  |  |
| Bourgogne-Franche-Comté | 64.0% | 1.2 | 0.8 | 1.7 |  |  | 37.1% | 1.1 | 0.7 | 1.6 |  |  |
| Normandie | 54.7% | 0.7 | 0.5 | 1.0 | * |  | 32.0% | 0.8 | 0.5 | 1.2 |  |  |
| Hauts-de-France | 59.3% | 0.9 | 0.6 | 1.3 |  |  | 33.7% | 0.9 | 0.7 | 1.3 |  |  |
| Grand-Est | 55.2% | 0.7 | 0.5 | 1.0 | * |  | 35.8% | 0.9 | 0.7 | 1.3 |  |  |
| Bretagne | 66.4% | 1.1 | 0.7 | 1.6 |  |  | 43.9% | 1.3 | 0.9 | 1.8 |  |  |
| Nouvelle Aquitaine | 65.2% | 1.0 | 0.7 | 1.4 |  |  | 34.5% | 0.8 | 0.6 | 1.2 |  |  |
| Occitanie | 61.8% | 0.8 | 0.6 | 1.1 |  |  | 41.6% | 1.1 | 0.8 | 1.5 |  |  |
| Auvergne-Rhône -Alpes | 63.5% | 0.9 | 0.7 | 1.2 |  |  | 35.4% | 0.8 | 0.6 | 1.1 |  |  |
| Provence-Alpes-Côte d’Azur | 62.1% | 0.8 | 0.6 | 1.1 |  |  | 36.7% | 1.0 | 0.7 | 1.3 |  |  |
| **Perceived level of information on the effects of diet on cancer risk** | | | | | |  |  |  |  |  |  |  |
| Very well informed (ref.) | 64.7% | 1 |  |  |  |  | 44.3% | 1 |  |  |  |  |
| Somewhat well informed | 65.9% | 1.0 | 0.8 | 1.2 |  |  | 39.3% | 0.7 | 0.6 | 0.9 | ** |  |
| Somewhat poorly informed | 60.2% | 0.7 | 0.6 | 0.9 | ** |  | 35.6% | 0.7 | 0.5 | 0.8 | *** |  |
| Very poorly informed | 53.2% | 0.7 | 0.5 | 0.9 | ** |  | 24.5% | 0.5 | 0.3 | 0.6 | *** |  |
| **Have you seen a general practitioner in the last 12 months?** | | | | | |  |  |  |  |  |  |  |
| Yes (ref.) | 62.9% | 1 |  |  |  |  | 38.2% | 1 |  |  |  |  |
| No | 60.5% | 1.0 | 0.8 | 1.1 |  |  | 32.2% | 0.8 | 0.7 | 1.0 |  |  |
| **Do you have or have you had cancer?** | | | | | |  |  |  |  |  |  |  |
| No (ref.) | 62.8% | 1 |  |  |  |  | 36.8% | 1 |  |  |  |  |
| Yes | 59.1% | 1.1 | 0.9 | 1.4 |  |  | 40.5% | 1.0 | 0.8 | 1.2 |  |  |
| **Do you feel personally at risk of developing cancer during your life?** | | | | | |  |  |  |  |  |  |  |
| Yes (ref.) | 65.7% | 1 |  |  |  |  | 38.2% | 1 |  |  |  |  |
| No | 56.4% | 0.8 | 0.7 | 0.9 | *** |  | 34.2% | 0.9 | 0.7 | 1.0 | * |  |
| **Has at least one of your relatives had cancer?** | | | | | |  |  |  |  |  |  |  |
| No (ref.) | 50.6% | 1 |  |  |  |  | 31.0% | 1 |  |  |  |  |
| Yes | 63.8% | 1.4 | 1.2 | 1.8 | ** |  | 37.7% | 1.0 | 0.8 | 1.3 |  |  |

|  | **Red meat (*n*=4613)**  **as a risk factor** | | | | |  | **Processed meat (*n*=4627)**  **as a risk factor** | | | | |  |
| --- | --- | --- | --- | --- | --- | --- | --- | --- | --- | --- | --- | --- |
|  | **Model 3** | | | | |  | **Model 4** | | | | |  |
|  | **%** | **OR** | **Lower limit 95% CI** | **Upper limit 95% CI** | ***p*** |  | **%** | **OR** | **Lower limit 95% CI** | **Upper limit 95% CI** | ***p*** |  |
| **Sex** |  |  |  |  |  |  |  |  |  |  |  |  |
| Male (ref.) | 64.6% | 1 |  |  |  |  | 74.1% | 1 |  |  |  |  |
| Female | 62.1% | 0.8 | 0.7 | 0.9 |  |  | 75.6% | 1.0 | 0.9 | 1.2 |  |  |
| **Age (years)** |  |  |  |  |  |  |  |  |  |  |  |  |
| 15-24 (ref.) | 58.6% | 1 |  |  |  |  | 67.9% | 1 |  |  |  |  |
| 25-34 | 67.7% | 1.4 | 1.0 | 1.8 |  |  | 76.2% | 1.1 | 0.8 | 1.5 |  |  |
| 35-44 | 64.7% | 1.3 | 1.0 | 1.7 |  |  | 71.3% | 0.9 | 0.7 | 1.2 |  |  |
| 45-54 | 64.5% | 1.4 | 1.1 | 1.8 |  |  | 77.7% | 1.4 | 1.1 | 1.9 | * |  |
| 55-64 | 65.7% | 1.5 | 1.2 | 2.0 |  |  | 78.6% | 1.5 | 1.1 | 2.1 | ** |  |
| 65-74 | 62.8% | 1.2 | 0.9 | 1.6 |  |  | 77.3% | 1.3 | 0.9 | 1.8 |  |  |
| 75-85 | 55.3% | 1.0 | 0.7 | 1.3 |  |  | 74.9% | 1.2 | 0.8 | 1.7 |  |  |
| **Occupation** | | | | | |  |  |  |  |  |  |  |
| Employee (ref.) | 60.4% | 1 |  |  |  |  | 71.3% | 1 |  |  |  |  |
| Tradesperson. storekeeper. self-employed worker. or farmer | 57.8% | 0.8 | 0.6 | 1.0 |  |  | 72.7% | 1.0 | 0.8 | 1.3 |  |  |
| Higher occupations | 75.6% | 1.3 | 1.0 | 1.6 |  |  | 85.4% | 1.4 | 1.1 | 1.9 | * |  |
| Intermediate occupations | 70.8% | 1.2 | 1.0 | 1.5 |  |  | 84.2% | 1.7 | 1.3 | 2.1 | *** |  |
| Working class | 52.5% | 0.7 | 0.6 | 0.8 | *** |  | 64.5% | 0.9 | 0.7 | 1.1 |  |  |
| Missing data | 62.2% | 1.4 | 1.0 | 1.8 | * |  | 70.3% | 1.3 | 0.9 | 1.7 |  |  |
| **Education level** |  |  |  |  |  |  |  |  |  |  |  |  |
| < High school (ref.) | 55.1% | 1 |  |  |  |  | 67.2% | 1 |  |  |  |  |
| High school | 67.7% | 1.5 | 1.3 | 1.8 | *** |  | 76.1% | 1.5 | 1.2 | 1.8 | *** |  |
| Postsecondary | 72.8% | 1.6 | 1.3 | 1.9 | *** |  | 85.6% | 2.2 | 1.8 | 2.7 | *** |  |
| **Tobacco consumption** | | | | | |  |  |  |  |  |  |  |
| No (ref.) | 64.7% | 1 |  |  |  |  | 76.9% | 1 |  |  |  |  |
| Yes | 59.8% | 0.8 | 0.7 | 0.9 | ** |  | 70.0% | 0.7 | 0.6 | 0.9 | *** |  |
| **Body mass index** | | | | | |  |  |  |  |  |  |  |
| 18.5-24.9 (ref.) | 54.3% | 1 |  |  |  |  | 68.1% | 1 |  |  |  |  |
| <18.5 | 66.6% | 0.6 | 0.5 | 0.9 | ** |  | 76.9% | 0.6 | 0.4 | 0.9 | ** |  |
| 25-29.9 | 61.3% | 0.8 | 0.7 | 0.9 | ** |  | 74.7% | 0.9 | 0.7 | 1.0 |  |  |
| >=30 | 59.9% | 0.8 | 0.7 | 1.0 | * |  | 72.1% | 0.8 | 0.7 | 1.0 |  |  |
| **Region** |  |  |  |  |  |  |  |  |  |  |  |  |
| Pays-de-la-Loire (ref.) | 65.8% | 1 |  |  |  |  | 81.4% | 1 |  |  |  |  |
| Ile-de-France (Paris region) | 68.1% | 1.1 | 0.8 | 1.4 |  |  | 75.6% | 0.7 | 0.5 | 1.0 |  |  |
| Centre-Val-de-Loire | 52.7% | 0.6 | 0.4 | 0.9 | * |  | 74.1% | 0.7 | 0.5 | 1.2 |  |  |
| Bourgogne-Franche-Comté | 62.3% | 1.0 | 0.6 | 1.4 |  |  | 74.5% | 0.8 | 0.5 | 1.3 |  |  |
| Normandie | 59.5% | 0.8 | 0.5 | 1.1 |  |  | 72.9% | 0.6 | 0.4 | 1.0 | * |  |
| Hauts-de-France | 58.7% | 0.8 | 0.6 | 1.1 |  |  | 68.9% | 0.6 | 0.4 | 0.8 | ** |  |
| Grand-Est | 57.4% | 0.7 | 0.5 | 1.0 |  |  | 75.6% | 0.8 | 0.5 | 1.1 |  |  |
| Bretagne | 70.1% | 1.2 | 0.8 | 1.7 |  |  | 85.8% | 1.3 | 0.8 | 2.1 |  |  |
| Nouvelle Aquitaine | 63.2% | 0.9 | 0.6 | 1.2 |  |  | 74.0% | 0.6 | 0.4 | 0.9 | * |  |
| Occitanie | 64.3% | 0.9 | 0.6 | 1.2 |  |  | 73.6% | 0.6 | 0.4 | 1.0 | * |  |
| Auvergne-Rhône -Alpes | 61.9% | 0.8 | 0.6 | 1.1 |  |  | 72.2% | 0.6 | 0.4 | 0.8 | ** |  |
| Provence-Alpes-Côte d’Azur | 66.2% | 1.0 | 0.7 | 1.4 |  |  | 75.6% | 0.7 | 0.4 | 1.0 |  |  |
| **Perceived level of information on the effects of diet on cancer risk** | | | | | |  |  |  |  |  |  |  |
| Very well informed (ref.) | 68.3% | 1 |  |  |  |  | 75.0% | 1 |  |  |  |  |
| Somewhat well informed | 65.1% | 0.8 | 0.6 | 0.9 | * |  | 78.4% | 1.0 | 0.8 | 1.3 |  |  |
| Somewhat poorly informed | 61.1% | 0.6 | 0.5 | 0.8 | *** |  | 72.5% | 0.7 | 0.6 | 0.9 | ** |  |
| Very poorly informed | 57.2% | 0.6 | 0.4 | 0.8 | *** |  | 67.8% | 0.7 | 0.5 | 1.0 | * |  |
| **Have you seen a general practitioner in the last 12 months?** | | | | | |  |  |  |  |  |  |  |
| Yes (ref.) | 65.0% | 1 |  |  |  |  | 76.6% | 1 |  |  |  |  |
| No | 55.4% | 0.7 | 0.6 | 0.8 | *** |  | 67.0% | 0.7 | 0.6 | 0.8 | *** |  |
| **Do you have or have you had cancer?** | | | | | |  |  |  |  |  |  |  |
| No (ref.) | 63.4% | 1 |  |  |  |  | 74.7% | 1 |  |  |  |  |
| Yes | 62.5% | 1.0 | 0.8 | 1.3 |  |  | 77.3% | 1.0 | 0.8 | 1.3 |  |  |
| **Do you feel personally at risk of developing cancer during your life?** | | | | | |  |  |  |  |  |  |  |
| Yes (ref.) | 67.5% | 1 |  |  |  |  | 79.0% | 1 |  |  |  |  |
| No | 55.9% | 0.7 | 0.6 | 0.8 | *** |  | 67.3% | 0.7 | 0.6 | 0.8 | *** |  |
| **Has at least one of your relative had cancer?** | | | | | |  |  |  |  |  |  |  |
| No (ref.) | 52.6% | 1 |  |  |  |  | 57.9% | 1 |  |  |  |  |
| Yes | 64.5% | 1.2 | 1.0 | 1.5 |  |  | 76.7% | 1.6 | 1.3 | 2.0 | *** |  |

**Legend:** * = *p*<.05; ** = *p*<.01; *** = *p<*.001; CI = confidence interval; OR = odds ratio; ref. = reference.

Models 1 to 4 are adjusted for: sex, age, occupation, education level, tobacco consumption, body mass index, region, perceived level of information, consultation with a general practitioner in the last 12 months, being or having been treated for cancer, feeling personally at risk of developing cancer and having at least one relative diagnosed with cancer.

**Supplementary Table 3. Factors associated with perception of the role of other specific foods in cancer risk.**

|  | **Pulses (*n*=4600)**  **as a protective factor** | | | | |  | **Organic foods (*n*=4888)**  **as a protective factor** | | | | |  | **Ultraprocessed foods (*n*=4627)**  **as a risk factor** | | | | |
| --- | --- | --- | --- | --- | --- | --- | --- | --- | --- | --- | --- | --- | --- | --- | --- | --- | --- |
|  | **Model 5** | | | | |  | **Model 6** | | | | |  | **Model 7** | | | | |
|  | **%** | **OR** | **Lower limit 95% CI** | **Upper limit 95% CI** | ***p*** |  | **%** | **OR** | **Lower limit 95% CI** | **Upper limit 95% CI** | ***p*** |  | **%** | **OR** | **Lower limit 95% CI** | **Upper limit 95% CI** | ***p*** |
| **Sex** |  |  |  |  |  |  |  |  |  |  |  |  |  |  |  |  |  |
| Male (ref.) | 45.2% | 1 |  |  |  |  | 52.7% | 1 |  |  |  |  | 89.3% | 1 |  |  |  |
| Female | 42.4% | 0.8 | 0.7 | 0.9 | ** |  | 53.4% | 0.9 | 0.8 | 1.1 |  |  | 88.6% | 0.8 | 0.6 | 1.0 | * |
| **Age (years)** |  |  |  |  |  |  |  |  |  |  |  |  |  |  |  |  |  |
| 15-24 (ref.) | 42.4% | 1 |  |  |  |  | 57.9% | 1 |  |  |  |  | 88.3% | 1 |  |  |  |
| 25-34 | 44.1% | 0.9 | 0.7 | 1.1 |  |  | 54.8% | 0.6 | 0.5 | 0.8 | *** |  | 91.6% | 1.1 | 0.7 | 1.8 |  |
| 35-44 | 41.2% | 0.8 | 0.6 | 1.1 |  |  | 54.2% | 0.6 | 0.5 | 0.8 | ** |  | 87.5% | 0.9 | 0.6 | 1.3 |  |
| 45-54 | 43.9% | 1.0 | 0.8 | 1.3 |  |  | 52.0% | 0.6 | 0.5 | 0.8 | *** |  | 86.0% | 0.7 | 0.5 | 1.1 |  |
| 55-64 | 48.8% | 1.2 | 1.0 | 1.6 |  |  | 53.1% | 0.8 | 0.6 | 1.0 |  |  | 93.8% | 1.9 | 1.2 | 3.0 | ** |
| 65-74 | 46.7% | 1.1 | 0.9 | 1.5 |  |  | 52.5% | 0.7 | 0.5 | 0.9 | * |  | 88.9% | 0.9 | 0.6 | 1.4 |  |
| 75-85 | 35.1% | 0.7 | 0.5 | 1.0 | * |  | 46.8% | 0.6 | 0.4 | 0.8 | ** |  | 84.9% | 0.7 | 0.4 | 1.1 |  |
| **Occupation** | | | | | |  |  |  |  |  |  |  |  |  |  |  |  |
| Employee (ref.) | 40.9% | 1 |  |  |  |  | 45.5% | 1 |  |  |  |  | 87.4% | 1 |  |  |  |
| Tradesperson. storekeeper. self-employed worker. or farmer | 37.8% | 0.8 | 0.6 | 1.1 |  |  | 49.8% | 1.1 | 0.9 | 1.5 |  |  | 87.9% | 0.9 | 0.6 | 1.3 |  |
| Higher occupations | 52.8% | 1.2 | 0.9 | 1.5 |  |  | 63.9% | 1.4 | 1.1 | 1.8 | ** |  | 94.7% | 1.2 | 0.8 | 1.9 |  |
| Intermediate occupations | 51.5% | 1.3 | 1.1 | 1.5 | * |  | 59.6% | 1.4 | 1.2 | 1.7 | *** |  | 93.8% | 1.5 | 1.0 | 2.1 | * |
| Working class | 36.2% | 0.8 | 0.7 | 1.0 |  |  | 40.1% | 1.0 | 0.8 | 1.2 |  |  | 80.7% | 0.6 | 0.5 | 0.9 | ** |
| Missing data | 41.8% | 1.0 | 0.7 | 1.3 |  |  | 56.4% | 1.3 | 1.0 | 1.8 | * |  | 90.9% | 1.8 | 1.2 | 2.9 | * |
| **Education level** |  |  |  |  |  |  |  |  |  |  |  |  |  |  |  |  |  |
| < High school (ref.) | 37.1% | 1 |  |  |  |  | 40.7% | 1 |  |  |  |  | 83.9% | 1 |  |  |  |
| High school | 46.5% | 1.4 | 1.2 | 1.7 | *** |  | 50.7% | 1.3 | 1.1 | 1.6 | *** |  | 92.1% | 1.8 | 1.3 | 2.4 | *** |
| Postsecondary | 51.9% | 1.6 | 1.4 | 1.9 | *** |  | 63.7% | 2.0 | 1.7 | 2.4 | *** |  | 94.7% | 2.3 | 1.7 | 3.2 | *** |
| **Tobacco consumption** | | | | | |  |  |  |  |  |  |  |  |  |  |  |  |
| No (ref.) | 45.3% | 1 |  |  |  |  | 54.3% | 1 |  |  |  |  | 89.4% | 1 |  |  |  |
| Yes | 39.9% | 0.8 | 0.7 | 0.9 | ** |  | 49.3% | 0.8 | 0.7 | 0.9 | ** |  | 87.8% | 0.9 | 0.7 | 1.2 |  |
| **Body mass index** | | | | | |  |  |  |  |  |  |  |  |  |  |  |  |
| 18.5-24.9 (ref.) | 43.7% | 1 |  |  |  |  | 55.7% | 1 |  |  |  |  | 89.8% | 1 |  |  |  |
| <18.5 | 45.0% | 1.1 | 0.8 | 1.5 |  |  | 58.2% | 0.8 | 0.6 | 1.2 |  |  | 90.6% | 0.8 | 0.5 | 1.4 |  |
| 25-29.9 | 43.2% | 0.9 | 0.8 | 1.0 |  |  | 49.0% | 0.7 | 0.6 | 0.8 | *** |  | 88.5% | 0.8 | 0.6 | 1.0 | * |
| >=30 | 41.5% | 0.9 | 0.8 | 1.1 |  |  | 44.6% | 0.6 | 0.5 | 0.8 | *** |  | 85.9% | 0.8 | 0.6 | 1.0 |  |
| **Region** |  |  |  |  |  |  |  |  |  |  |  |  |  |  |  |  |  |
| Pays-de-la-Loire (ref.) | 46.0% | 1 |  |  |  |  | 54.2% | 1 |  |  |  |  | 90.0% | 1 |  |  |  |
| Ile-de-France (Paris region) | 46.8% | 1.0 | 0.7 | 1.3 |  |  | 56.2% | 0.9 | 0.7 | 1.2 |  |  | 85.7% | 0.5 | 0.3 | 0.9 | * |
| Centre-Val-de-Loire | 38.9% | 0.8 | 0.5 | 1.2 |  |  | 50.2% | 0.9 | 0.6 | 1.3 |  |  | 81.4% | 0.4 | 0.2 | 0.8 | ** |
| Bourgogne-Franche-Comté | 42.7% | 0.9 | 0.6 | 1.4 |  |  | 55.3% | 0.9 | 0.6 | 1.3 |  |  | 85.6% | 0.8 | 0.4 | 1.6 |  |
| Normandie | 39.3% | 0.8 | 0.6 | 1.2 |  |  | 48.9% | 0.7 | 0.5 | 1.0 | * |  | 89.2% | 0.8 | 0.4 | 1.6 |  |
| Hauts-de-France | 41.9% | 1.0 | 0.7 | 1.3 |  |  | 48.9% | 0.7 | 0.5 | 1.0 |  |  | 86.1% | 0.7 | 0.4 | 1.3 |  |
| Grand-Est | 42.2% | 0.9 | 0.7 | 1.3 |  |  | 47.5% | 0.8 | 0.6 | 1.1 |  |  | 90.4% | 1.0 | 0.6 | 1.9 |  |
| Bretagne | 47.8% | 1.0 | 0.7 | 1.5 |  |  | 59.1% | 1.1 | 0.8 | 1.6 |  |  | 93.2% | 1.3 | 0.6 | 2.5 |  |
| Nouvelle Aquitaine | 42.5% | 0.8 | 0.6 | 1.1 |  |  | 51.8% | 0.9 | 0.6 | 1.2 |  |  | 93.0% | 1.2 | 0.7 | 2.1 |  |
| Occitanie | 45.5% | 1.0 | 0.7 | 1.3 |  |  | 54.5% | 0.9 | 0.6 | 1.2 |  |  | 94.4% | 1.7 | 0.9 | 3.2 |  |
| Auvergne-Rhône -Alpes | 42.6% | 0.8 | 0.6 | 1.1 |  |  | 55.7% | 0.9 | 0.6 | 1.2 |  |  | 87.6% | 0.7 | 0.4 | 1.1 |  |
| Provence-Alpes-Côte d’Azur | 43.7% | 0.8 | 0.6 | 1.2 |  |  | 48.7% | 0.8 | 0.6 | 1.1 |  |  | 91.0% | 1.0 | 0.5 | 1.7 |  |
| **Perceived level of information on the effects of diet on cancer risk** | | | | | |  |  |  |  |  |  |  |  |  |  |  |  |
| Very well informed (ref.) | 47.9% | 1 |  |  |  |  | 56.5% | 1 |  |  |  |  | 84.6% | 1 |  |  |  |
| Somewhat well informed | 46.8% | 0.9 | 0.8 | 1.1 |  |  | 54.6% | 1.0 | 0.8 | 1.2 |  |  | 90.1% | 1.4 | 1.0 | 1.8 |  |
| Somewhat poorly informed | 39.3% | 0.7 | 0.6 | 0.9 | ** |  | 52.1% | 0.9 | 0.7 | 1.2 |  |  | 90.7% | 1.5 | 1.1 | 2.1 | * |
| Very poorly informed | 40.5% | 0.8 | 0.6 | 1.1 |  |  | 44.8% | 0.9 | 0.7 | 1.1 |  |  | 83.6% | 1.0 | 0.7 | 1.5 |  |
| **Have you seen a general practitioner in the last 12 months?** | | | | | |  |  |  |  |  |  |  |  |  |  |  |  |
| Yes (ref.) | 44.1% | 1 |  |  |  |  | 53.6% | 1 |  |  |  |  | 90.1% | 1 |  |  |  |
| No | 42.3% | 1.0 | 0.8 | 1.1 |  |  | 50.4% | 0.8 | 0.7 | 1.0 | * |  | 83.6% | 0.7 | 0.5 | 0.8 | ** |
| **Do you have or have you had cancer?** | | | | | |  |  |  |  |  |  |  |  |  |  |  |  |
| No (ref.) | 43.6% | 1 |  |  |  |  | 53.2% | 1 |  |  |  |  | 88.8% | 1 |  |  |  |
| Yes | 45.3% | 0.9 | 0.7 | 1.2 |  |  | 51.6% | 1.0 | 0.8 | 1.3 |  |  | 90.9% | 0.8 | 0.5 | 1.2 |  |
| **Do you feel personally at risk of developing cancer during your life?** | | | | | |  |  |  |  |  |  |  |  |  |  |  |  |
| Yes (ref.) | 45.6% | 1 |  |  |  |  | 56.0% | 1 |  |  |  |  | 92.1% | 1 |  |  |  |
| No | 39.7% | 0.8 | 0.7 | 1.0 | * |  | 46.8% | 0.8 | 0.7 | 0.9 | *** |  | 83.9% | 0.6 | 0.5 | 0.8 | *** |
| **Has at least one of your relatives had cancer?** | | | | | |  |  |  |  |  |  |  |  |  |  |  |  |
| No (ref.) | 42.0% | 1 |  |  |  |  | 41.5% | 1 |  |  |  |  | 74.3% | 1 |  |  |  |
| Yes | 43.9% | 0.9 | 0.8 | 1.2 |  |  | 54.0% | 1.4 | 1.1 | 1.7 | ** |  | 90.6% | 2.4 | 1.8 | 3.2 | *** |

|  | **Dietary supplements (*n*=4731)**  **as having no influence** | | | | |  | **Skipping meals (*n*=2431)**  **as having no influence** | | | | |  | **Dairy products (*n*=4593)**  **as having no influence** | | | | |  |  |  |  |  |
| --- | --- | --- | --- | --- | --- | --- | --- | --- | --- | --- | --- | --- | --- | --- | --- | --- | --- | --- | --- | --- | --- | --- |
|  | **Model 8** | | | | |  | **Model 9** | | | | |  | **Model 10** | | | | |  |  |  |  |  |
|  | **%** | **OR** | **Lower limit 95% CI** | **Upper limit 95% CI** | ***p*** |  | **%** | **OR** | **Lower limit 95% CI** | **Upper limit 95% CI** | ***p*** |  | **%** | **OR** | **Lower limit 95% CI** | **Upper limit 95% CI** | ***p*** |  |  |  |  |  |
| **Sex** |  |  |  |  |  |  |  |  |  |  |  |  |  |  |  |  |  |  |  |  |  |  |
| Male (ref.) | 44.8% | 1 |  |  |  |  | 27.2% | 1 |  |  |  |  | 64.7% | 1 |  |  |  |  |  |  |  |  |
| Female | 59.5% | 2.0 | 1.7 | 2.3 | ******* |  | 23.5% | 0.8 | 0.7 | 1.0 |  |  | 70.7% | 1.5 | 1.3 | 1.7 | *** |  |  |  |  |  |
| **Age (years)** |  |  |  |  |  |  |  |  |  |  |  |  |  |  |  |  |  |  |  |  |  |  |
| 15-24 (ref.) | 46.9% | 1 |  |  |  |  | 34.7% | 1 |  |  |  |  | 62.3% | 1 |  |  |  |  |  |  |  |  |
| 25-34 | 47.0% | 0.9 | 0.7 | 1.2 |  |  | 31.8% | 1.1 | 0.7 | 1.6 |  |  | 64.4% | 0.9 | 0.7 | 1.2 |  |  |  |  |  |  |
| 35-44 | 55.1% | 1.3 | 1.0 | 1.7 | ***** |  | 25.0% | 0.8 | 0.5 | 1.1 |  |  | 68.9% | 1.0 | 0.8 | 1.3 |  |  |  |  |  |  |
| 45-54 | 54.2% | 1.3 | 1.0 | 1.7 | ***** |  | 27.1% | 1.0 | 0.7 | 1.5 |  |  | 68.4% | 1.1 | 0.8 | 1.4 |  |  |  |  |  |  |
| 55-64 | 53.8% | 1.4 | 1.1 | 1.9 | ****** |  | 22.7% | 0.6 | 0.4 | 0.9 | * |  | 69.3% | 1.1 | 0.8 | 1.5 |  |  |  |  |  |  |
| 65-74 | 55.4% | 1.5 | 1.1 | 2.0 | ****** |  | 18.8% | 0.6 | 0.4 | 0.9 | * |  | 69.9% | 1.1 | 0.8 | 1.5 |  |  |  |  |  |  |
| 75-85 | 54.8% | 1.6 | 1.2 | 2.3 | ****** |  | 19.4% | 0.6 | 0.4 | 1.0 |  |  | 74.4% | 1.6 | 1.1 | 2.2 | * |  |  |  |  |  |
| **Occupation** | | | | | |  |  |  |  |  |  |  |  |  |  |  |  |  |  |  |  |  |
| Employee (ref.) | 56.7% | 1 |  |  |  |  | 24.7% | 1 |  |  |  |  | 67.2% | 1,0 |  |  |  |  |  |  |  |  |
| Tradesperson. storekeeper. self-employed worker. or farmer | 46.9% | 0.9 | 0.7 | 1.1 |  |  | 29.1% | 1.6 | 1.1 | 2.4 | * |  | 70.2% | 1.3 | 1.0 | 1.7 | * |  |  |  |  |  |
| Higher occupations | 50.0% | 1.0 | 0.8 | 1.3 |  |  | 23.0% | 0.8 | 0.6 | 1.2 |  |  | 66.0% | 1.2 | 0.9 | 1.5 |  |  |  |  |  |  |
| Intermediate occupations | 53.2% | 1.1 | 0.9 | 1.3 |  |  | 24.9% | 0.8 | 0.6 | 10.1 |  |  | 68.2% | 1.2 | 0.9 | 1.4 |  |  |  |  |  |  |
| Working class | 50.5% | 1.2 | 1.0 | 1.5 |  |  | 23.8% | 0.9 | 0.6 | 1.2 |  |  | 69.4% | 1.4 | 1.1 | 1.7 | ** |  |  |  |  |  |
| Missing data | 47.3% | 0.7 | 0.6 | 1.0 | ***** |  | 33.3% | 1.3 | 0.9 | 1.9 |  |  | 62.4% | 0.8 | 0.6 | 1.0 |  |  |  |  |  |  |
| **Education level** |  |  |  |  |  |  |  |  |  |  |  |  |  |  |  |  |  |  |  |  |  |  |
| < High school (ref.) | 51.3% | 1 |  |  |  |  | 22.3% | 1 |  |  |  |  | 68.9% | 1 |  |  |  |  |  |  |  |  |
| High school | 53.5% | 1.2 | 1.0 | 1.4 | ***** |  | 27.8% | 1.1 | 0.8 | 1.4 |  |  | 66.4% | 1.0 | 0.8 | 1.1 |  |  |  |  |  |  |
| Postsecondary | 53.5% | 1.1 | 0.9 | 1.3 |  |  | 26.2% | 1.3 | 1.1 | 1.7 |  |  | 67.3% | 1.0 | 0.8 | 1.2 |  |  |  |  |  |  |
| **Tobacco consumption** | | | | | |  |  |  |  |  |  |  |  |  |  |  |  |  |  |  |  |  |
| No (ref.) | 54.8% | 1 |  |  |  |  | 24.6% | 1 |  |  |  |  | 68.7% | 1 |  |  |  |  |  |  |  |  |
| Yes | 51.4% | 0.9 | 0.8 | 1.0 |  |  | 27.0% | 1.0 | 0.8 | 1.2 |  |  | 69.4% | 1.1 | 0.9 | 1.3 |  |  |  |  |  |  |
| **Body mass index** | | | | | |  |  |  |  |  |  |  |  |  |  |  |  |  |  |  |  |  |
| 18.5-24.9 (ref.) | 54.8% | 1 |  |  |  |  | 32.2% | 1 |  |  |  |  | 68.7% | 1 |  |  |  |  |  |  |  |  |
| <18.5 | 55.4% | 0.9 | 0.7 | 1.2 |  |  | 24.8% | 1.6 | 1.0 | 2.5 |  |  | 65.8% | 0.9 | 0.7 | 1.3 |  |  |  |  |  |  |
| 25-29.9 | 51.5% | 0.9 | 0.8 | 1.0 |  |  | 25.0% | 1.0 | 0.8 | 1.2 |  |  | 71.1% | 1.2 | 1.0 | 1.4 | * |  |  |  |  |  |
| >=30 | 55.5% | 1.1 | 0.9 | 1.3 |  |  | 24.4% | 0.9 | 0.7 | 1.2 |  |  | 65.2% | 0.9 | 0.8 | 1.1 |  |  |  |  |  |  |
| **Region** |  |  |  |  |  |  |  |  |  |  |  |  |  |  |  |  |  |  |  |  |  |  |
| Pays-de-la-Loire (ref.) | 48.6% | 1 |  |  |  |  | 33.6% | 1 |  |  |  |  | 63.8% | 1 |  |  |  |  |  |  |  |  |
| Ile-de-France (Paris region) | 49.9% | 1.3 | 1.0 | 1.7 |  |  | 26.6% | 0.6 | 0.4 | 1.0 |  |  | 62.4% | 1.1 | 0.8 | 1.4 |  |  |  |  |  |  |
| Centre-Val-de-Loire | 57.4% | 1.4 | 1.0 | 2.1 |  |  | 33.0% | 0.9 | 0.5 | 1.7 |  |  | 72.8% | 1.6 | 1.0 | 2.4 | * |  |  |  |  |  |
| Bourgogne-Franche-Comté | 49.1% | 1.0 | 0.7 | 1.4 |  |  | 19.2% | 0.5 | 0.3 | 0.9 | * |  | 65.7% | 1.0 | 0.7 | 1.5 |  |  |  |  |  |  |
| Normandie | 54.4% | 1.5 | 1.0 | 2.1 | * |  | 20.0% | 0.5 | 0.3 | 0.9 | * |  | 74.1% | 1.8 | 1.2 | 2.7 | ** |  |  |  |  |  |
| Hauts-de-France | 55.2% | 1.3 | 0.9 | 1.8 |  |  | 23.5% | 0.6 | 0.4 | 1.0 |  |  | 65.9% | 1.1 | 0.8 | 1.5 |  |  |  |  |  |  |
| Grand-Est | 50.6% | 1.2 | 0.9 | 1.7 |  |  | 21.5% | 0.6 | 0.3 | 1.0 | * |  | 69.3% | 1.4 | 1.0 | 2.0 | * |  |  |  |  |  |
| Bretagne | 53.9% | 1.4 | 1.0 | 2.1 |  |  | 26.0% | 0.7 | 0.4 | 1.3 |  |  | 73.4% | 1.6 | 1.1 | 2.4 | * |  |  |  |  |  |
| Nouvelle Aquitaine | 50.4% | 1.2 | 0.9 | 1.7 |  |  | 24.9% | 0.6 | 0.4 | 1.0 | * |  | 66.1% | 1.2 | 0.8 | 1.6 |  |  |  |  |  |  |
| Occitanie | 57.5% | 1.6 | 1.1 | 2.2 | ** |  | 24.3% | 0.7 | 0.4 | 1.1 |  |  | 69.4% | 1.4 | 1.0 | 1.9 |  |  |  |  |  |  |
| Auvergne-Rhône -Alpes | 53.5% | 1.3 | 1.0 | 1.8 |  |  | 22.3% | 0.6 | 0.4 | 0.9 | * |  | 73.3% | 1.7 | 1.2 | 2.4 | ** |  |  |  |  |  |
| Provence-Alpes-Côte d’Azur | 51.3% | 1.2 | 0.8 | 1.7 |  |  | 26.3% | 0.7 | 0.4 | 1.2 |  |  | 66.7% | 1.1 | 0.8 | 1.6 |  |  |  |  |  |  |
| **Perceived level of information on the effects of diet on cancer risk** | | | | | |  |  |  |  |  |  |  |  |  |  |  |  |  |  |  |  |  |
| Very well informed (ref.) | 50.9% | 1 |  |  |  |  | 28.0% | 1 |  |  |  |  | 58.9% | 1 |  |  |  |  |  |  |  |  |
| Somewhat well informed | 53.8% | 1.1 | 0.9 | 1.4 |  |  | 25.0% | 1.0 | 0.8 | 1.4 |  |  | 69.0% | 1.6 | 1.3 | 1.9 | *** |  |  |  |  |  |
| Somewhat poorly informed | 52.6% | 1.1 | 0.9 | 1.4 |  |  | 24.4% | 0.9 | 0.7 | 1.3 |  |  | 70.9% | 1.7 | 1.4 | 2.1 | *** |  |  |  |  |  |
| Very poorly informed | 48.2% | 0.9 | 0.7 | 1.2 |  |  | 25.6% | 0.9 | 0.6 | 1.4 |  |  | 63.0% | 1.3 | 1.0 | 1.7 |  |  |  |  |  |  |
| **Have you seen a general practitioner in the last 12 months?** | | | | | |  |  |  |  |  |  |  |  |  |  |  |  |  |  |  |  |  |
| Yes (ref.) | 52.3% | 1 |  |  |  |  | 25.5% | 1 |  |  |  |  | 67.8% | 1 |  |  |  |  |  |  |  |  |
| No | 52.7% | 1.2 | 1.0 | 1.4 | * |  | 24.0% | 0.8 | 0.6 | 1.0 |  |  | 67.9% | 1.1 | 0.9 | 1.3 |  |  |  |  |  |  |
| **Do you have or have you had cancer?** | | | | | |  |  |  |  |  |  |  |  |  |  |  |  |  |  |  |  |  |
| No (ref.) | 52.3% | 1 |  |  |  |  | 25.7% | 1 |  |  |  |  | 68.0% | 1 |  |  |  |  |  |  |  |  |
| Yes | 53.9% | 1.1 | 0.9 | 1.4 |  |  | 20.4% | 1.2 | 0.8 | 1.7 |  |  | 66.7% | 1.3 | 1.0 | 1.7 | * |  |  |  |  |  |
| **Do you feel personally at risk of developing cancer during your life?** | | | | | |  |  |  |  |  |  |  |  |  |  |  |  |  |  |  |  |  |
| Yes (ref.) | 51.1% | 1 |  |  |  |  | 25.8% | 1 |  |  |  |  | 66.6% | 1 |  |  |  |  |  |  |  |  |
| No | 55.0% | 1.1 | 1.0 | 1.3 |  |  | 24.2% | 1.0 | 0.8 | 1.3 |  |  | 71.0% | 1.2 | 1.1 | 1.4 | ** |  |  |  |  |  |
| **Has at least one of your relatives had cancer** | | | | | |  |  |  |  |  |  |  |  |  |  |  |  |  |  |  |  |  |
| No (ref.) | 52.1% | 1 |  |  |  |  | 32.0% | 1 |  |  |  |  | 67.4% | 1 |  |  |  |  |  |  |  |  |
| Yes | 54.1% | 0.9 | 0.7 | 1.1 |  |  | 24.7% | 0.7 | 0.5 | 0.9 | * |  | 68.9% | 1.0 | 0.8 | 1.3 |  |  |  |  |  |  |

**Legend:** * = *p*<.05; ** = *p*<.01; *** = *p*<.001; CI = confidence interval; OR = odds ratio; ref. = reference.

Models 5 to 10 are adjusted for: sex, age, occupation, education level, tobacco consumption, body mass index, region, perceived level of information, consultation with a general practitioner in the last 12 months, being or having been treated for cancer, feeling personally at risk of developing cancer and having at least one relative diagnosed with cancer.
